# Identifying post stroke patients in the Malaysian community: Profile of patients managed at ten selected public health centres in Peninsular Malaysia

**DOI:** 10.1186/1471-2458-14-S1-P20

**Published:** 2014-01-29

**Authors:** Aznida Firzah Abdul Aziz, Noor Azah Abd Aziz, Saperi Sulong, Syed Mohamed Aljunid

**Affiliations:** 1 UNU IIGH, Universiti Kebangsaan Malaysia, UKM Medical Centre Complex, Jalan Yaacob Latif, 56000 Cheras, Kuala Lumpur, Malaysia; 2 Jabatan Kesihatan Masyarakat, Pusat Perubatan Universiti Kebangsaan Malaysia, Jalan Yaacob Latif, Bandar Tun Razak, 56000 Cheras, Kuala Lumpur, Malaysia

## Background

Post stroke patients are managed either at hospital-based specialist outpatient or primary care clinics after acute stroke. There is scarce local data on patients receiving treatment at primary care after hospital discharge. This study aimed to determine the profile of post stroke patients managed at public health centres in Peninsular Malaysia.

## Materials and methods

Practice registry lists at ten public health centres were screened for patients with cerebrovascular accident (CVA) between July and December 2012. Patients aged ≥18 years with recorded diagnosis of CVA either radiographically or by referring physician were recruited. Patients with transient ischaemic attack, traumatic brain injury or isolated nerve palsies were excluded. Details of stroke risk factors and clinical findings at first primary care visit were extracted from case notes and from patient/and carer interviews. Data were analysed for mean, median and proportions.

## Results

Total of 121 patients were recruited. Mean age at stroke was 56.3 (SD ±10.1) years, with 78.7% at ≤5 years post-stroke. Median duration between acute stroke and contact with primary care team was 160 (0-10564) days. Main cause of stroke was ischaemia (73%) followed by haemorrhage (8%). For stroke risk factors: 87.9% had hypertension, 76.5% had type 2 diabetes mellitus, 85.3% dyslipidaemia and 8% were current smokers. Target blood pressure control achieved for diabetics (≤130/80mmHg) was 30.2% and non-diabetic patients (≤ 140/90mmHg) 47.8%. Mean total cholesterol was 5.1 (SD ±1.1), LDL-cholesterol 3.0 (SD ±0.9), HDL-cholesterol 1.2 (SD ±0.3) and median triglyceride level 1.5 (0.5-7.2) mmol/L. It was found that 77.3% had undergone rehabilitation with median duration 5.3 (0.3-6) months for physiotherapy and 8.0 (3-6) months for occupational therapy. For functional status, 47.6% had moderate to total dependence.

## Conclusions

Post stroke patients at primary care were younger than expected. Contact with primary care after discharge from hospital should be expedited to optimise treatment of risk factors, especially blood pressure control. Occupational therapy was the predominant type of rehabilitation utilised.

